# Genomic Insights into *Candidozyma auris* Clade II: Comparative Phylogenomics and Structural Validation of Fluconazole Resistance Mechanisms

**DOI:** 10.3390/jof12010076

**Published:** 2026-01-20

**Authors:** Sanghak Lee, Kei-Anne Garcia Baritugo, Han-Soo Kim, Hyeyoung Lee, Sook Won Ryu, Soo-Young Kim, Chae Hoon Lee, Young Ree Kim, Jeong Hwan Shin, Jayoung Kim, Gi-Ho Sung

**Affiliations:** 1 Biomedical Institute of Mycological Resource, International St. Mary’s Hospital, College of Medicine, Catholic Kwandong University, Incheon 22711, Republic of Korea; leesh762@cku.ac.kr (S.L.); 645099@ish.ac.kr (K.-A.G.B.); 2 Department of Biomedical Sciences, Graduate School, Catholic Kwandong University, Gangneung 25601, Republic of Korea; hansk@cku.ac.kr; 3 Department of Laboratory Medicine, International St. Mary’s Hospital, College of Medicine, Catholic Kwandong University, Incheon 22711, Republic of Korea; shomermaid@ish.ac.kr; 4 Department of Laboratory Medicine, School of Medicine, Kangwon National University, Chuncheon 24331, Republic of Korea; ryusw@kangwon.ac.kr; 5 Department of Laboratory Medicine, College of Medicine, Catholic University of Korea, St. Vincent’s Hospital, Suwon 16247, Republic of Korea; vsykim@catholic.ac.kr; 6 Department of Laboratory Medicine, College of Medicine, Yeungnam University, Daegu 42415, Republic of Korea; chlee@med.yu.ac.kr; 7 Department of Laboratory Medicine, College of Medicine, Jeju National University, Jeju 63243, Republic of Korea; namu8790@jejunu.ac.kr; 8 Department of Laboratory Medicine, College of Medicine, Inje University, Busan 47392, Republic of Korea; jhsmile@paik.ac.kr; 9 Department of Convergence Science, College of Medicine, Catholic Kwandong University, Gangneung 25601, Republic of Korea

**Keywords:** *Candidozyma auris*, *Candida auris*, fluconazole resistance, comparative genomics, whole-genome sequencing, in silico structural modeling, phylogenomics, clade II, regulatory modulation

## Abstract

*Candidozyma auris* (formerly *Candida auris*) is an emerging multidrug-resistant fungal pathogen with confirmed cases in over 30 countries. Although whole-genome sequencing (WGS) analysis defined distinct clades during characterization of underlying genetic mechanism behind multidrug resistance, Clade II remains under-evaluated. In this study, a three-level comparative genomic strategy (Global, Clade, Phenotype) was employed by integration of unbiased genome-wide comparative SNP screening (GATK v4.1.9.0), targeted BLAST profiling (BLAST+ v2.17.0), and in silico protein analysis (ColabFold v1.5.5; DynaMut2 v2.0) for systematic evaluation of mechanisms of antifungal resistance in thirty-nine Clade II *C. auris* clinical isolates and fourteen reference strains. Global and clade-level analyses confirmed that all the clinical isolates belong to Clade II, according to phylogenetic clustering and mating type locus (*MTL*) conservation. At the phenotype level, a distinct subclade of fluconazole-resistant mutants was identified to have a heterogenous network of mutations in seven key enzymes associated with cell membrane dynamics and the metabolic stress response. Among these, four core mutations (*TAC1B*, *CAN2*, *NIC96*, *PMA1*) were confirmed as functional drivers based on strict criteria during multitier in silico protein analysis: cross-species conservation, surface exposure, active site proximity, thermodynamic stability, and protein interface interaction. On the other hand, three high-level fluconazole-resistant clinical isolates (≥128 μg/mL) that lacked these functional drivers were subjected to comprehensive subtractive genomic profiling analysis. The absence of coding mutations in validated resistance drivers, yeast orthologs, and convergent variants suggests that there is an alternative novel non-coding or regulatory mechanism behind fluconazole resistance. These findings highlight Clade II’s evolutionary divergence into two distinct trajectories towards the development of a high level of fluconazole resistance: canonical protein alteration versus regulatory modulation.

## 1. Introduction

*Candidozyma auris* (formerly *Candida auris*) is an emerging multidrug-resistant fungal pathogen associated with health care-associated infections in the bloodstream (candidiasis/fungemia), wounds, urinary tract, ear (otitis media or externa), bones (osteomyelitis), brain (meningitis), and/or heart lining (pericarditis) [1]. The name was recently updated based on its phylogenetic divergence, intrinsic antifungal resistant behavior, and distinct growth pattern that separates it from other Candida species such as *Candida haemulonii*, *Candida pseudohaemulonii*, *Candida albicans*, *Candida tropicalis*, and *Candida parapsilosis* [2]. *C. auris* was firstly identified in Japan, after it was isolated from a patient with otitis externa [3], but the first documented case of *C. auris* was in South Korea, where it was isolated from 15 patients with chronic otitis media between 2004 and 2006. However, it was misidentified as *Candida haemulonii* [4,5]. Since then, *C. auris*-related infection has been reported in over 45 countries across six continents [6]. High mortality and morbidity rates (29–62%) have been associated with *C. auris*, but the exact rates vary according to the clinical management of infection, patient’s underlying conditions, age, as well as geographic region [7,8]. In addition to its prevalence, the propensity of *C. auris* to develop resistance to commonly used classes of antifungal drugs such as azoles (fluconazole, voriconazole, posaconazole, itraconazole), polyene (amphotericin B, nystatin), echinocandins (micafungin, anidulafungin, caspofungin), and nucleoside analog (5-fluorocytosine) has raised significant concerns in clinical practice [1,7].

The global emergence of antifungal-resistant strains demands the development of novel therapeutic strategies. However, this requires the characterization of the genetic basis for antifungal resistance. To this end, the epidemiology and genomic sequence of antifungal-resistant *C. auris* were characterized by single-nucleotide polymorphism (SNP) analysis based on whole-genome sequencing (WGS) data and antifungal susceptibility testing (AFST). SNP-based WGS analysis revealed that *C. auris* isolates grouped into six distinct clades according to the geographic region, and genetic divergence between clades was observed, together with significant genetic homogeneity or clonal expansion within each clade [9,10].

Besides a unique geographic origin, further comparative WGS analysis indicates that the mating type locus (*MTL*) is also distinct between clades. The *MTL* is a genomic region in fungi that determines its mating type and its ability to undergo sexual reproduction. In diagnostics, characterization of the highly conserved *MTL* region helps track outbreaks of *C. auris* and allows for precise clade-specific identification. Recently, *MTL* of *C. auris* isolates from Clades I to IV have been characterized and clade-specific conservation of *MTL* idiomorphs (*MTLa*, *MTLα*) was confirmed, with *MTLa* present in Clades I, and IV, while *MTLα* was present in Clades II and III [11]. For recently discovered Clades V and VI, *MTLa* and *MTLα* were observed, respectively [10,12,13,14].

On the other hand, AFST and SNP-based WGS analyses have also revealed that the antifungal susceptibility profiles and subsequent genetic basis of antifungal resistance significantly vary between clades. Recently, a systematic comparison of antifungal resistance patterns across all six clades was conducted by an analysis antifungal susceptibility profiles of 1031 strains described in 50 studies for the identification of clades with higher resistance rates against fluconazole, amphotericin B, and echinocandins (micafungin, anidulafungin, caspofungin). Clades I, III, and IV exhibit variable resistance to amphotericin B and echinocandins but collectively they have the highest percentage of fluconazole resistance, with 94%, 96%, and 44%, respectively. Meanwhile, Clades II, V, and VI are clades with higher susceptibility to antifungal agents. However, there is a very limited percentage of isolates evaluated for Clades II, V, and VI (0.3–3.2%), as compared to Clades I, III, and IV (30–33.3%) [13,14,15]. Across all clades, fluconazole resistance (77.1%) was the most prevalent followed by amphotericin B (24.2%), caspofungin (9.2%), micafungin (4.4%), and anidulafungin (1.7%) [15]. Fluconazole resistance is widely used as a first-line treatment of fungal infections because of its oral bioavailability, broad-spectrum activity, and low cost. However, the prolonged and repeated exposure to fluconazole has resulted in the emergence of resistant strains with molecular mechanisms based on mutations and/or duplication of key enzymes in critical pathways [16]. The main mechanism of action of fluconazole is the competitive binding inhibition of lanosterol 14α-demethylase (ERG11), which blocks the synthesis of ergosterol, a critical sterol involved in fungal cell membrane integrity and function. Fluconazole disrupts the fluidity and function of the fungal cell membrane, which ultimately leads to toxic sterol accumulation and cell death. In fluconazole-resistant isolates, the two main mechanisms of fluconazole resistance observed are as follows: (1) reduction in fluconazole binding affinity because of point mutations in *ERG11*, and (2) decreased intracellular drug levels because of upregulation of efflux pumps, major facilitator superfamily type efflux pumps (MDR1), and ATP-binding cassette type efflux pump (CDR1), caused by mutations in their associated transcription factors, which are *MRR1A* and *TAC1* or their variants *TAC1A* or *TAC1B*, respectively [17,18,19]. Among several mutations identified in key proteins, the following mutations show consistent pattern in multiple isolates across different studies: (1) Clade I—*ERG11*: K143R, Y132F and *TAC1B*: A640V; (2) Clade II—*ERG11*: K143R, Q357K, Y132F, L43H, and *TAC1B*: F214S, R495G; (3) Clade III—MRR1A: N647T, and *ERG11*: F126L; (4) Clade IV—*ERG11*: Y132F, F444L, and *TAC1B*: S611P; and (5) Clade V—*ERG11*: Y132F and *TAC1B*: D599G [12,13,15,20,21,22,23].

Compared to Clades I, III, and IV, fluconazole resistance in Clade II is less understood because most isolates are susceptible to fluconazole, which limits the availability of WGS data available for resistance characterization. Among 13 Clade II isolates with available WGS data, only 3 isolates are resistant to fluconazole: (1) TWCC 58362 from Japan, (2) B11809, and (3) B11808 from Gwangju, South Korea [24,25]. Previously, Clade II isolates were mostly susceptible to fluconazole, and mutations in *ERG11*, *TAC1A*, and *TAC1B* were rare and inconsistent [9,20,26]. However, recently, several fluconazole resistant Clade II isolates reportedly exhibit moderate fluconazole resistance (4–64 mg/L MIC), and several mutations in *ERG11*, *TAC1A*, and *TAC1B* have been identified [20,24]. However, experimental validations of these mutations are also sparse compared to progress achieved with Clade I, II, and III studies. This disparity highlights the need to conduct more comparative genomic studies, and functional characterization experiments to gain deeper insight into the mechanism of fluconazole resistance in Clade II isolates.

In this study, a comprehensive three-level comparative genomic strategy (Global, Clade, and Phenotype) was employed to gain deeper knowledge regarding antifungal resistance mechanisms in under-evaluated Clade II strains. Whole-genome sequences of fifty-three strains were analyzed and utilized in this study, including thirty-nine clinical isolates from South Korea and fourteen global reference strains (thirteen Clade II and one Clade I). Firstly, genome-wide phylogenomic analysis was used to reconstruct the evolutionary history and establish the placement of the clinical isolates at a global level. Then, *MTL* conservation was assessed to confirm clade lineage stability and verify the absence of inter-clade recombination at the clade level. Finally, at the phenotype level, a multi-tiered strategy was applied to decipher the genetic basis of antifungal resistance. Firstly, an unbiased genome-wide comparative single-nucleotide polymorphism (SNP) screening analysis was used to identify potential novel resistance drivers across the entire coding genome of all strains analyzed in this study. After identification of potential enzymes and their associated mutations, these candidates were distinguished as functional drivers versus neutral hitchhikers by subjection to rigorous multi-tier in silico structural analysis pipeline: (1) structural model and stability prediction using AlphaFold2/ColabFold and DynaMut2 for generation of high-confidence 3D models and quantification of thermodynamic impact of mutations on enzyme stability, (2) evolutionary conservation analysis across fungal orthologs (*Candida albicans*, *Saccharomyces cerevisiae*) for filtration of mutations in highly conserved and functionally critical residues, (3) functional proximity analysis for the assessment of the spatial distance of mutations to relative active sites or ligand binding pockets (<10 Å), (4) residue accessibility analysis for distinction of surface-active residues from those buried in hydrophobic core, and (5) interface interaction analysis for the evaluation of potential disruption of essential protein-protein interaction domains. Alternatively, for high-level resistant strains that lacked the structurally validated drivers identified in this study, a systematic subtractive genomic analysis was conducted to investigate alternative non-coding or regulatory mechanisms.

## 2. Materials and Methods

### 2.1. Clinical Sample Isolation, Culture, and Identification

Thirty-nine *C. auris* isolates were collected from patients across six South Korean hospitals between 2016 and 2020. The specific region of South Korea (SK) and hospital where isolates were obtained from are as follows: (1) Jeju, SK—Jeju National University Hospital, (2) Gangwon, SK—Kangwon National University Hospital, (3) Incheon, SK—International St. Mary’s Hospital, (4) Gyeonggi, SK—St. Vincent’s Hospital, (5) Daegu, SK—Yeungnam University Hospital, and (6) Busan, SK—Busan Paik Hospital. For epidemiological analysis, patient data such as demographics, infection sites, and clinical outcomes were obtained from hospital records. Strains were primarily isolated from ear discharge and ear swabs. Isolates were cultured on Yeast Malt (YM) agar at 30 °C for 1–2 days. Species identification was conducted by amplification of Internal Transcribed Spacer (ITS) region and Large Subunit region of the ribosomal RNA (LSU or 28s rRNA) using universal primers ITS1F/ITS4 and LR0/LR5 (Macrogen, Seoul, Republic of Korea), respectively. Detailed description of isolates are listed in Supplementary Table S1.

### 2.2. Antifungal Susceptibility Testing (AFST)

Antifungal susceptibility was evaluated based on the M27-ED4 broth microdilution method in accordance with the Clinical and Laboratory Standards Institute Standard Guide 4th Edition [27]. Each well of a 96-well plate was inoculated with 2.5 × 10^3^ *C. auris* conidia and incubated at 35 °C. After 24 h of incubation, Minimum Inhibitory Concentration (MICs) were determined as the drug concentrations that achieved 100% growth inhibition compared to an antifungal-free control. The quality control strain used was *Candida albicans* ATCC 14053 (American Type Culture Collection, Manassas, VA, USA). Tentative MIC breakpoints used to define susceptibility according to Centers for Disease Control and Prevention (CDC) standards [27] are as follows: anidulafungin ≥ 4 μg/mL, micafungin ≥ 4 μg/mL, caspofungin ≥ 2 μg/mL, amphotericin B ≥ 2 μg/mL, and fluconazole ≥ 64 μg/mL [28,29]. Additional breakpoints were evaluated based on established values for other related Candida species due to a lack of *C. auris*-specific breakpoints for the corresponding antifungal agents as follows: 5-flucytosine ≥ 128 μg/mL, voriconazole ≥ 4 μg/mL, itraconazole ≥ 1 μg/mL, posaconazole ≥ 4 μg/mL [29,30,31]. Resistance profiles were classified based on identified MIC values and correlated with genetic variants identified in the proceeding section for downstream genetic analysis of the resistance mechanism. Fluconazole resistance, the primary focus of this study, was stratified into three phenotypic categories: susceptible (MIC < 64 μg/mL), resistant (MIC 64–127 μg/mL), and high-level resistant (MIC ≥ 128 μg/mL). Clinical isolates were classified as fluconazole-resistant mutants (FRMs) if MIC ≥ 64 μg/mL. Resistance profiles were correlated with genetic variants identified through subsequent genomic and in silico analyses.

### 2.3. Whole-Genome Sequencing, Genome Assembly, and Single-Nucleotide Polymorphism Variant Analysis

Genomic DNA of *C. auris* isolates were extracted using a DNeasy PowerSoil Kit (Qiagen, Hilden, Germany), according to the manufacturer’s protocol. Sequencing libraries were prepared using the Nextera XT DNA Library Prep Kit (Illumina, San Diego, CA, USA). Subsequently, sequencing was conducted using the HiSeq X platform (Illumina) for generation of 150 bp paired-end reads. Whole-genome sequencing (WGS) data for 14 global reference strains were retrieved from the Sequence Read Archive (SRA) database (National Center for Biotechnology Information [NCBI], Bethesda, MD, USA). These included: (1) fluconazole-susceptible Clade I representative isolate (B8441) from Pakistan, (2) 1 fluconazole-susceptible Clade II representative isolate (B11220) from Japan, (3) 3 fluconazole-resistant Clade II representative strains from Japan (TWCC 58362) and Gwangju, Republic of Korea (B11808, B11809), and (4) 9 fluconazole-susceptible Clade II representative strains from Canada (B13463), the United States of America or USA (B14308, B12081, B12043), and Japan (TWCC 50952, TWCC 58191, TWCC 13878, TWCC 13847, TWCC 13846) [9,25,26].

The quality of raw reads was assessed using FastQC v0.11.0 [32]. Fastp v0.20.1 and Cutadapt v1.18 were used to filter reads for the removal of adapters and retention of sequences with Phred quality scores ≥ 20 and a minimum length ≥ 30 bp [33,34]. Filtered reads were aligned with reference genomes for Clade I representative *C. auris* B8441 (GenBank: GCA_002759435.2) and Clade II representative *C. auris* B11220 (GenBank: GCA_003013715.2), using BWA mem v0.7.17 [9,11]. Variants such as SNPs and indels were identified using GA TK v4.1.9.0 in haploid mode, with the following filters: quality by depth (QD) < 2.0, Fisher’s strand bias (FS) > 60.0, mapping quality (MQ) < 40.0, minimum depth ≥ 10, and allele frequency ≥ 0.8 [35]. SnpEff v5.0e was used to annotate the function [36]. For the identification of novel mutations, identified variants were cross-referenced with a custom database of resistance-associated SNPs. The raw sequencing reads and assembled WGS sequences that correspond to reported isolates have been deposited in the NCBI Sequence Read Archive (SRA) and GenBank, under BioProject PRJNA1343880. The associated BioSample and assembled WGS sequence accessions numbers are SAMN52628946–SAMN52628908 and JBRTRJ000000000–JBRTSV000000000, respectively (Supplementary Table S1).

### 2.4. Global-Level Phylogenomic Analysis

Phylogenomic analysis was performed by alignment of 28,956 SNPs from 52 *C. auris* strains with B11220, a Clade II reference genome. Clinical isolates from this study were compared to thirteen confirmed Clade II strains from Japan, the United States of America, and Canada, for the assessment of regional genetic patterns and epidemiological relationships. Maximum likelihood phylogenetic trees were generated using FastTree v2.1.10 with 1000 bootstrap iterations for robustness [37]. Interactive Tree of Life (iTOL v6) was used for visualization of the phylogenetic tree [38]. Clade I strain B8441 was used as an outgroup to contextualize Clade II diversity.

### 2.5. Clade-Level Validation via Mating Type Locus (MTL)

The *MTL* (*MTLa* or *MTLα*) of isolates were analyzed for the assessment of locus preservation and genetic stability. WGS data were used for identification of *MTL* alleles (*MTLa1*, *MTLa2*, *MTLα1*, *MTLα2*) by alignment of sequencing reads to *MTL* regions of reference genomes B8441 (Clade I) and B11220 (Clade II) using BWA mem v0.7.17. Variants were identified using GATK v4.1.9.0. SnpEff v5.0e was used for the annotation of functional impacts of detected SNPs and indels. Integrative Genomics Viewer (IGV v2.16) was used for the analysis of read coverage and detection of deletions or rearrangements. Confirmation of *MTL* preservation was conducted by the examination of WGS data for allele presence and sequence integrity. Allele frequencies were analyzed for the assessment of sample purity and detection of potential mixed-strain populations. *MTL* stability was evaluated by the identification of disruptive mutations such as frameshift and stop codons. Cross-breeding risk was assessed by the comparison of cross-clade *MTLα* for Clade II with *MTLa* from Clades I and IV.

### 2.6. Unbiased Genome-Wide Comparative SNP Screening

Based on phylogenomic clustering and AFST results, the Clade II population was differentiated into a distinct “fluconazole-resistant mutant” (FRM) subclade (MIC ≥ 128 μg/mL) and a susceptible wild-type group (MIC < 64 μg/mL). Then, these groups were subjected to comprehensive, unbiased, genome-wide comparative SNP screening to identify potential novel resistance drivers. Variants were filtered for the identification of non-synonymous mutations, which were as follows: (1) uniquely enriched in FRM subclade, (2) absent in susceptible clinical isolates, and (3) located in genes with predicted enzymatic or transport functions. Genome-wide scanning was used to identify a distinct mutational network that would be subsequently prioritized for targeted profiling.

### 2.7. Targeted Resistance Gene Profiling

After the unbiased genome-wide comparative SNP screening, targeted mutational profiling was conducted on the identified candidates for a precise characterization of genotype–phenotype correlations. The candidates from unbiased screening are as follows: *TAC1B* (transcription factor), *PMA1* (plasma membrane H^+^-ATPase), *NIC96* (nuclear pore scaffold), *CAN2* (arginine permease), *EXO70* (exocyst component), *PCK1* (gluconeogenesis), and *VPS53* (vacuolar sorting). Additionally, classical azole and echinocandin resistance genes were screened to rule out known mechanisms: *ERG11* (lanosterol 14-α demethylase; azole target), *FKS1* (1,3-β-glucan synthase; echinocandin target), *CDR1*, and *MDR1* (efflux transporters). Mutations were validated if they met an allele frequency ≥ 0.8 and depth ≥ 10 × thresholds. Mutation penetrance was calculated as the proportion of FRM isolates carrying the variant.

### 2.8. Multi-Tier In Silico Structural Validation

A rigorous five-tier structural analysis pipeline was used to distinguish functional drivers of fluconazole resistance from neutral hitchhikers among the targeted candidates. Firstly, three-dimensional structures of wild-type and mutant proteins were created and predicted using AlphaFold2 via ColabFold (ColabFold v1.5.5). Structures with pLDDT confidence scores > 70 in the mutation region were retained for analysis. Then, the created protein models were analyzed using DynaMut2 v2.0 to quantify thermodynamic stability changes (ΔΔG, kcal/mol). Mutations with Gibbs free energy (ΔΔG) scores were classified as follows: (1) >1.0 kcal/mol—destabilizing or loss-of-function, (2) −1.0 to 1.0 kcal/mol—neutral, and (3) <−1.0 kcal/mol—stabilizing or gain-of-function. Subsequently, all candidates were subjected to four additional evolutionary and spatial protein analyses, regardless of stability results. Cross-species conservation of mutated residues was quantified by global alignment of candidate proteins against orthologs from *Saccharomyces cerevisiae*, *Candida glabrata*, and *Aspergillus fumigatus*. Structural proximity analysis was conducted to measure the Euclidean distances between mutated residues and functional sites (active sites, metal coordination centers, binding interfaces, and protein centroid). Surface residue accessibility analysis was performed for differentiation of structural core mutations from surface-exposed variations by enumeration of Cα atoms within an 8 Å radius (neighbor count <15 = surface-exposed, ≥15 = buried). Buried residues were prioritized as potential drivers since core destabilization is a primary mechanism of resistance in this dataset. Meanwhile, highly exposed surface residues were identified as neutral polymorphisms, except where the involvement in protein–protein interfaces was observed. For enzymes with functions related to obligate multi-subunit assemblies or oligomers (NIC96, EXO70, VPS53, TAC1B), protein–protein interaction was characterized by analyzing predicted interface residues using 3D structure geometry and co-evolutionary constraints. Mutations were identified as functional drivers of fluconazole resistance if they met ≥ 3 of 5 criteria: (1) evolutionary cross-species conservation (≥67%), (2) low surface accessibility (buried) or interface involvement, (3) active site/binding site proximity (<10 Å), (4) thermodynamic stability change (ΔΔG > 1.0), and (5) protein interface involvement. Mutations meeting < 3 criteria were classified as neutral hitchhikers or clonal variants with no significant impact on fluconazole resistance.

### 2.9. Mystery Strain Characterization: Subtractive Genomic Profiling, Ortholog Discovery, and Functional Annotation

A specialized subtractive genomic profiling approach was employed for “mystery strains”, clinical isolates which have high-level fluconazole resistance (MIC ≥ 128 μg/mL) and a validated lack of identified functional drivers. This strategy was employed for the identification of non-coding or regulatory mechanisms by isolation of variants unique to this high-resistance phenotype. Firstly, the variant set of all strains (susceptible vs. resistant) was computationally subtracted from the genomic profiles of the identified mystery strains. Then, the remaining variants were systematically filtered for prioritization of the following traits: (1) Uniqueness: absent in all other susceptible and resistant strains, (2) High-Frequency: allele frequency ≥ 0.8, (3) Functional Potential: variants were prioritized if located in putative regulatory regions (upstream gene variants/5’-UTRs within 1000 bp) or coding regions (non-synonymous/indel) according to SnpEff annotations, and (4) Convergent: presence across phylogenetically distinct mystery strains. Estimation of random mutation convergence was evaluated using a Poisson distribution model, wherein the expected number of independent recurrence events (*E*) was calculated based on the rate of background mutation and total analyzable genome size. To ensure that the identified targets represented independent evolutionary acquisitions instead of clonal inheritance, convergence was only considered statistically significant at *p* < 10^−12^. For inference of the biological function of the novel fluconazole resistance drivers in mystery strains, a targeted BLAST profiling of extracted protein sequences was conducted against proteomes of model fungal organisms: *Saccharomyces cerevisiae* S288C, *Candida glabrata* CBS 138, and *Aspergillus fumigatus* Af293. Orthologs were designated according to the Reciprocal Best Hits (RBH) based on the following strict criteria, E-value ≤ 10^−5^ and sequence identity ≥ 25%. InterProScan v5.0 and Pfam v34.0 were used for mapping functional domains. UniProt databases were used for cross-referencing annotations. High-confidence orthologs were designated according to conservation (≥67%) in critical domains (active sites, DNA-binding motifs) or complete orthology (RBH E-value < 10^−30^). This functional context was used for categorization of candidates into mechanistic classes (e.g., transporters, transcription factors, signaling kinases) for the evaluation of effect of non-coding or regulatory mechanisms on modulation of fluconazole resistance in the mystery strains.

### 2.10. Statistical Analysis

Fisher’s exact test was used for the evaluation of non-random associations between resistance phenotypes (Resistant vs. Susceptible) and specific genetic variants. One-way analysis of variance (ANOVA) was used for the analysis of differences in antifungal susceptibility distributions across identified genomic subgroups on log_2_-transformed MIC values. Subsequently, Tukey’s HSD post hoc test was used for pairwise comparisons of significant main effects. Pearson’s correlation coefficient (*r*) was calculated for the assessment of linear relationship between the cumulative mutation burden (number of mutated enzymes per isolate) and degree of fluconazole resistance (log_2_ MIC). The Chi-square test was used for the assessment of differences in mutation frequencies between subclades. Benjamini–Hochberg (FDR) analysis was used to adjust *p*-values to control false discovery rates during genome-wide screening of mutated enzymes that can be candidate drivers of fluconazole resistance. All *p*-values were two-tailed, with statistical significance defined as *p* < 0.05. R v4.0.5 was used for all statistical analyses, and ggplot2 v3.3.0 was used for the generation of images for data visualization.

## 3. Results

### 3.1. Clinical Characteristics, Global Phylogenomic Placement, and Clade Lineage Stability

**Figure 1:**
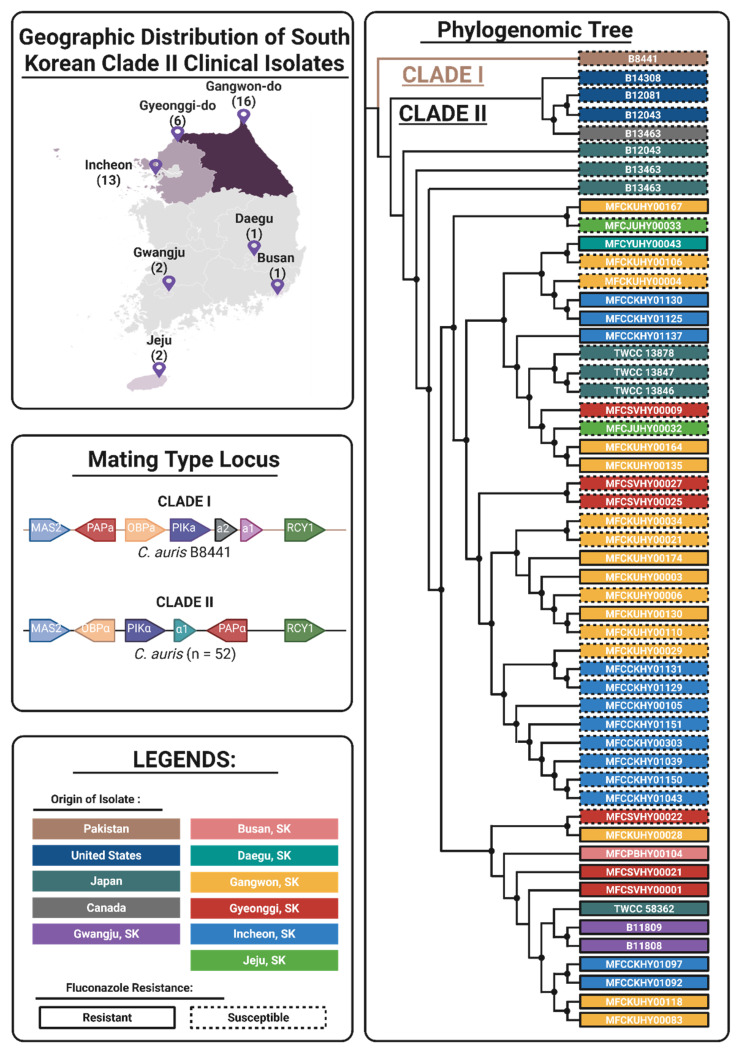
Global phylogenomic placement and clade lineage stability of Clade II clinical isolates: Geographic distribution of 39 South Korean clinical isolates with number of isolates per region described in parenthesis. Schematic comparison of *MTL* conservation between Clade I and Clade II strains. Enzymes: PIK1—phosphatidylinositol kinase, OBP1—oxysterol binding protein, PAP1—poly(A) polymerase. Maximum-likelihood phylogenomic tree constructed from 28,956 genome-wide SNPs.

Thirty-nine *C. auris* strains were isolated from ear-related specimens (abscess, discharge, swag) collected from six South Korean hospitals between the years 2016 and 2020 (Supplementary Table S1). The isolates were distributed across the following regions of South Korea: Busan (1), Daegu (1), Jeju (2), Gyeonggi (6), Incheon (13), and Gangwon (16) (Figure 1). Similar to the established clinical profile of the East Asian lineage, the isolates were primarily recovered from ear-related specimens (otitis media/externa) rather than invasive bloodstream infections. To establish the global phylogenomic placement and evolutionary context of these clinical isolates, a maximum-likelihood phylogenomic tree was reconstructed based on 28,956 genome-wide SNPs. All South Korean clinical isolates clustered robustly with the other thirteen global Clade II reference strains (Japan, USA, Canada). The formation of a monophyletic lineage distinct from the Clade I outgroup (B8441) confirms that the recent clinical cases in South Korea are clonally derived from the localized East Asian Clade II lineage rather than the recent introduction of multidrug-resistant Clade I or III strains (Figure 1). WGS analysis of clinical isolates confirmed the conservation of the *MTLa* locus in a representative Clade I strain and the *MTLα* locus in all 52 Clade II strains (39 collected clinical isolates + 13 NCBI reference strains), including *OBPα*, *PIKα*, *α1*, and *PAPα* (Figure 1). Phylogenetic analysis also demonstrates the clear separation between Clade I and Clade II, which suggests that there was no genetic recombination or cross-breeding. This is consistent with previous reports, where *MTLα* is consistently conserved among Clade II isolates [10,11].

### 3.2. Phenotypic Stratification

According to the CLSI guidelines, the antifungal susceptibility of thirty-nine *C. auris* isolates was tested against the following antifungal agents: (1) Azoles—Fluconazole, Itraconazole, Voriconazole, Posaconazole, (2) Polyene—Amphotericin B, (3) Echinocandins—Caspofungin, Micafungin, Anidulafungin, and (4) Nucleoside analog—Flucytosine or 5-Flucytosine (Table 1). No resistance was observed for amphotericin B (≥2 µg/mL), caspofungin (≥2 µg/mL), anidulafungin (≥4 µg/mL), micafungin (≥4 µg/mL), and 5-fluorocytosine (≥128 µg/mL). For azoles, no resistance was observed for itraconazole (≥1 µg/mL), voriconazole (≥4 µg/mL), and posaconazole (≥4 µg/mL). The only antifungal resistance observed was for fluconazole, where 46% of clinical isolates showed a MIC between 32 and 128 µg/mL (Table 1). Statistical clustering of MIC values identified two distinct phenotypic groups. There is a susceptible/intermediate baseline group, which is composed of clinical isolates that displayed low to moderate MICs (≤64 μg/mL). This group represents wild-type susceptible strains or early adaptive Clade II background strains. A high-level fluconazole-resistant group composed of nine strains was also observed with MICs above 128 μg/mL. This stratification highlights the emergence of a specific sub-population of Clade II isolates that have evolved to have a high level of fluconazole resistance. Thus, the genome of this subclade was subjected to comprehensive genomic profiling for elucidation of the mechanism of fluconazole resistance.

### 3.3. Unbiased Discovery of the “FRM” Resistance Network

**Figure 2:**
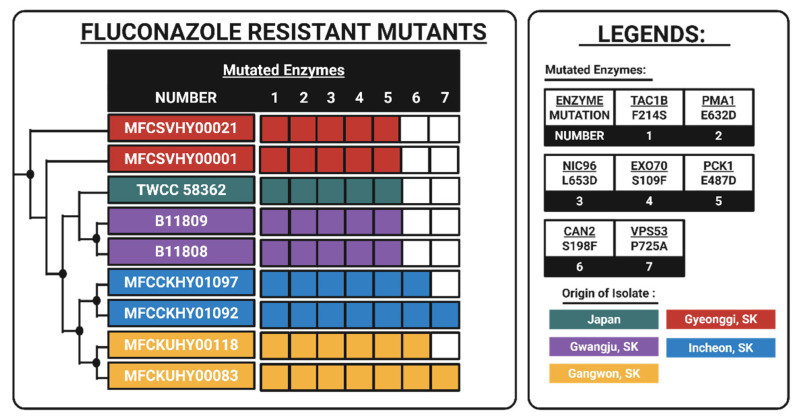
Mutational heatmap of the “FRM” resistance network. The heatmap displays the presence (solid color) or absence (white) of non-synonymous mutations in the seven key enzymes identified by genome-wide screening.

An unbiased genome-wide comparative SNP screening analysis was conducted to determine if the high-level resistant group shared a common genetic basis of fluconazole resistance. This genomic analysis revealed significant differentiation within the phenotypically uniform resistant cluster. As depicted in Figure 2, there are six isolates under the fluconazole-resistant mutant subclade, which are distinguished by a network of non-synonymous mutations, accumulated in stepwise manner.

On the other hand, there are three isolates, named mystery strains (MFCPBHY00104, MFCKUHY00167, MFCYUHY00043) because despite displaying a high level of fluconazole resistance, these strains did not exhibit any of the mutations observed in the FRM group. These strains will be analyzed further in later sections of this paper. Focusing on the FRM group, there was a unique enrichment of non-synonymous mutations in key enzymes associated with upstream regulation, cell membrane dynamics, and metabolic stress response (Table 2). Fisher’s exact test confirmed the significant association between the seven key enzymes’ signature and the fluconazole-resistant phenotype of the FRM group (*p* < 0.001) Interestingly, there were no mutations observed in the *ERG11* locus, the established primary driver of fluconazole resistance in Clade I. This confirms that fluconazole resistance displayed by the FRM subclade is driven by a non-canonical network that does not involve direct drug target modification.

### 3.4. Stepwise Accumulation and Dosage-Dependent Resistance

Targeted mutational profiling of the 39 clinical isolates revealed that resistance in the canonical FRM subclade is driven by a stepwise accumulation of mutation instead of development of a single polymorphism. Regression analysis indicated that there is a significant positive correlation (*R* = 0.69, *p* < 0.0001) between the cumulative mutation burden and fluconazole MIC, which supports a dosage-dependent evolutionary model.

**Figure 3:**
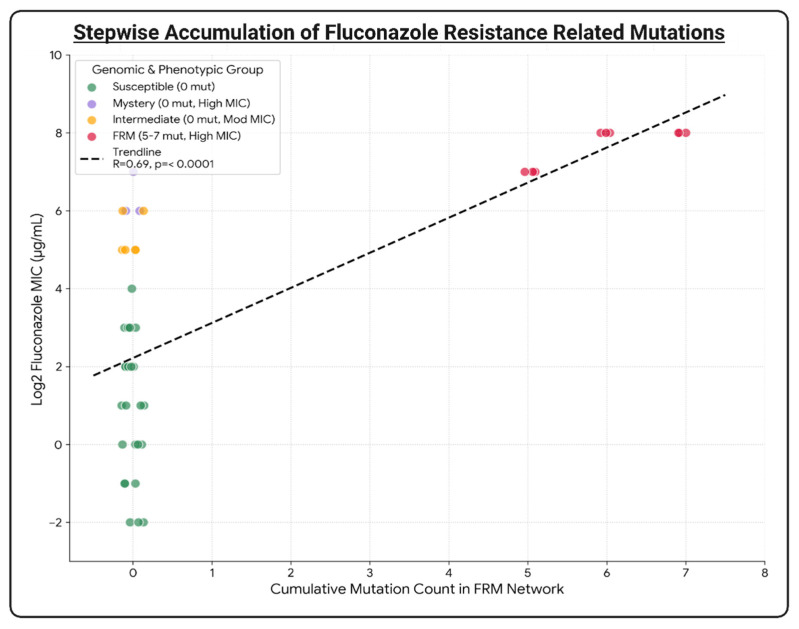
Stepwise evolution of resistance: Scatter plot showing the correlation (*r* = 0.69) between mutation count (X-axis) and fluconazole MIC (Y-axis).

In addition, the analysis identified a specific 0 → 5 → 6 → 7 accumulation pattern, as depicted in Figure 3. The wild-type baseline depicts a diverse phenotypic mix of susceptible, intermediate, and mystery strains because of a shared lack of mutations in the identified network in the preceding section. On the other hand, the acquisition of a resistance network demonstrates a strict threshold-based trajectory, with a high level of fluconazole resistance only emerging after the simultaneous accumulation of five mutations in the core network (*TAC1B*, *PMA1*, *NIC96*, *EXO70*, *PCK1*), as seen in FRM core subclade. Subsequently, a transitional state was observed with sequential expansion to six mutations by the additional accumulation of *CAN2* variants. Finally, the maximum mutations, seven, was observed after the addition of *VPS53* mutations. This stepwise trajectory indicates that five mutations in the core network serve as a critical evolutionary tipping point, after which subsequent additional mutations are recruited for stabilization of a high level of the fluconazole resistance phenotype.

### 3.5. Structural Validation of Functional Drivers

For strict differentiation between key drivers of fluconazole resistance and neutral polymorphisms or hitchhikers within the identified seven-gene network present in the FRM group, a multi-tier in silico structural validation pipeline was used for evaluation of the following key biophysical criteria: (1) thermodynamic stability (∆∆G), (2) evolutionary conservation, (3) active site proximity, (4) surface exposure, and (5) interface impact (Table 3). The enzyme is considered a validated functional driver if it passes ≥3 out 5 criteria.

**Figure 4:**
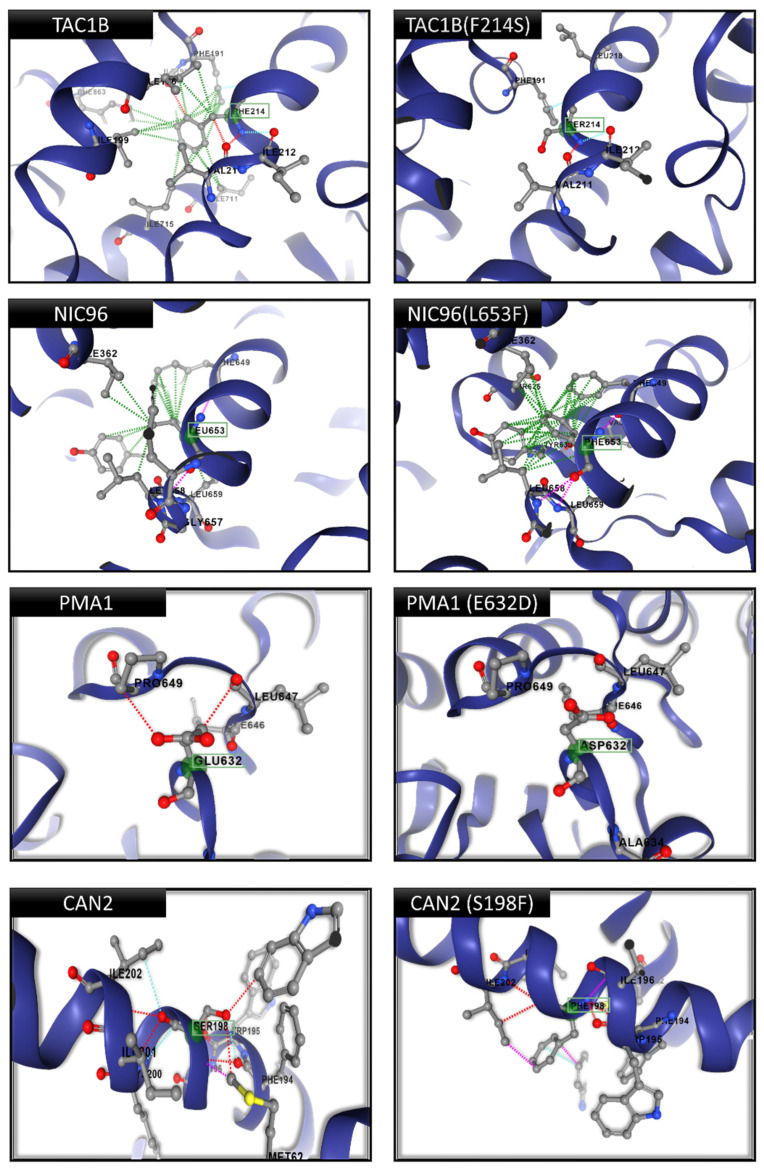
Structural mechanism of validated resistance drivers. Protein structural modeling (AlphaFold2/DynaMut2) compares the wild-type (left panels) and mutant (right panels) models to reveal the molecular impact of four key driver mutations: *TAC1B* (F214S): Destabilization of the dimerization interface (∆∆G = −3.81 kcal/mol); *PMA1* (E632D): Disruption of hydrogen bonding loops essential for membrane anchoring; *NIC96* (L653F): Steric overpacking within the nuclear pore complex hinge region; and *CAN2* (S198F): Pore occlusion caused by the introduction of a bulky phenylalanine residue. Legend: The wild-type backbone is colored gray. Mutant residues are colored by atom type (Red: Oxygen, Blue: Nitrogen). Key residues are highlighted in green and identified by labels in green boxes. Dotted lines represent atomic interactions: green dotted lines indicate hydrogen bonds, while red/pink dotted lines indicate steric clashes or repulsive interactions.

Based on Table 3, four mutations were validated as functional drivers. Firstly, for transcription factor *TAC1B*, the F214S mutation replaces a large hydrophobic phenylalanine with a small polar serine (Figure 4). Protein modeling revealed that this substitution creates a significant hydrophobic “void” (∆∆G = −3.81 kcal/mol) at the dimer interface, which destabilizes the quaternary structure that is essential for its repressor activity. Similarly, the *PMA1* (E632D) mutation in the plasma membrane H^+^-ATPase results in a “shortening defect”, where the aspartic acid side chain fails to maintain critical hydrogen bonding with the adjacent loop (distance > 6 Å) (Figure 4). This loss of interaction likely disrupts the pump’s membrane anchoring mechanism and potentially alters pH homeostasis. On the other hand, for the nuclear pore complex component *NIC96*, the L653F mutation introduces a bulky phenylalanine ring into a restricted hydrophobic pocket (Figure 4). This substitution causes severe steric overpacking, which is predicted to increase local rigidity and impair the flexibility required for nuclear transport. Finally, the *CAN2* (S198F) mutation involves the substitution of a small serine with a massive phenylalanine at the helical interface of the nutrient permease (Figure 4). This change generates severe steric clashes with Isoleucine 202. This would likely distort the transporter channel and affect nutrient uptake signaling. In contrast, the mutations identified in *EXO70* (S109F), *PCK1* (E487D), and *VPS53* (P725A) failed to meet the score threshold for functional drivers. Structural analysis placed these variants in non-conserved, surface-exposed regions where they exhibited a negligible impact on protein stability (∆∆G > −0.5 kcal/mol) and no interference with active sites or interfaces. Consequently, these were classified as “hitchhikers,” likely co-selected during the clonal expansion of the resistant lineage rather than directly contributing to the resistance mechanism.

### 3.6. Evidence for Non-Coding Mechanisms in High-Level Resistant Isolates

**Figure 5:**
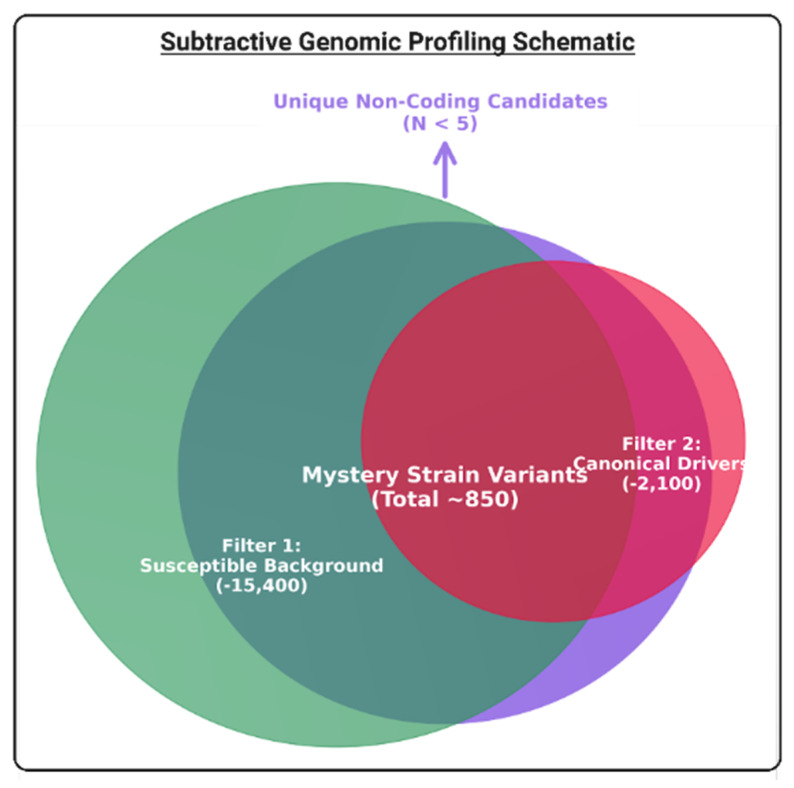
Subtractive genomic profiling of non-coding resistance: Venn diagram summarizing the subtractive filtering strategy used for characterization of the three mystery strains (MFCPBHY00104, MFCKUHY00167, MFCYUHY00043).

Despite the strong genotype–phenotype correlation observed in the canonical FRM group, the three high-level resistant “mystery” isolates (MFCPBHY00104, MFCKUHY00167, MFCYUHY00043) presented a paradoxical genomic profile. Although their phenotypic resistance was statistically indistinguishable from the FRM group (MIC ≥ 128 μg/mL), they clustered with the susceptible baseline in the phylogenomic tree and stepwise mutation analysis (Figure 1 and Figure 3). These strains possessed zero coding mutations in the validated seven-gene network, which indicates that there could be an alternative mechanism of fluconazole resistance. To address this, comprehensive subtractive genomic profiling was conducted for the identification of unique variants in this specific lineage. Filtration of the mystery strain genomes against the pan-genome of the all the other clinical isolates (both susceptible and canonical resistant) revealed that zero unique candidate mutations existed within the coding regions of any known resistance-associated genes (Figure 5). The complete absence of unique coding drivers strongly implies that resistance in the three mystery strains could be driven by non-coding mechanisms. These would likely involve epigenetic modulation or distal regulatory variants in untranslated regions (UTRs), which could upregulate efflux activity or stress responses without an alteration of protein structures, thereby representing a distinct evolutionary trajectory from the canonical mutational network in the FRM group.

## 4. Discussion

*Candidozyma auris* (*Candida auris*) has established itself as a global nosocomial pathogen, characterized by persistence in hospital environments and rapid development of multidrug resistance. The genomic mechanisms driving resistance in Clade I (South Asian) and Clade III (African) are well-documented, typically involving specific point mutations in the ergosterol biosynthetic pathway [16]. However, the evolutionary and genomic mechanism behind antifungal resistance in the East Asian Clade II lineage has remain less studied. Historically and in this study, Clade II isolates are from external ear canals, and most isolates are susceptible to all classes of systemic antifungal drugs, as was true of the isolates in this study [9,10]. Recently high levels of fluconazole resistance in Clade II strains have been reported, and in this study, 46% of clinical isolates were resistant to fluconazole, with nine strains demonstrating a MIC ≥ 128 μg/mL (Table 1) [20,24,26]. These recent reports indicate that Clade II can evolve to have high-level fluconazole resistance through complex, non-canonical mechanisms distinct from the globally dominant clones. Thus, in this study, 39 clinical isolates from South Korean hospitals and 14 reference strains were used for a comprehensive WGS-based comparative genomic analysis for the evaluation and identification of the genomic mechanism behind a high level of fluconazole resistance in Clade II isolates.

Firstly, phylogenomic analysis of the South Korean cohort confirmed that all isolates formed a monophyletic lineage nested within the global Clade II diversity based on Clade II representative strains from the USA, Canada, and Japan. All Clade II isolates were found to be distinct from the Clade I outgroup. This phylogeographic placement is clinically significant; unlike the invasive outbreaks often driven by Clade I, the isolation of these strains primarily from otitis media and externa cases reinforces the strong tropism of Clade II for the auditory canal [3,4,24]. The exclusive isolation of these strains from ear-related specimens highlights the unique ecological niche of *C. auris* Clade II. Unlike Clade I, which is frequently associated with invasive candidemia and thermotolerance at high temperatures, Clade II isolates have historically demonstrated temperature sensitivity, growing optimally at the cooler temperatures found in the external auditory canal (~37 °C or lower) but showing reduced survival at higher temperatures (>40 °C). Consistent with their phylogenomic placement in this lineage, the high-level resistant isolates in this study are predicted to retain this characteristic biological restriction. This suggests that while these isolates have evolved resistance to topical or systemic fluconazole, they have not yet adapted to survive the higher thermal stress associated with invasive systemic infections. Furthermore, the complete conservation of the *MTLα* idiomorph across the cohort supports a model of strictly clonal expansion rather than sexual recombination. This genetic stability implies that the emergence of high-level resistance likely arose through de novo mutation accumulation within a stable genetic background rather than through the acquisition of resistance determinants via recent cross-clade breeding events. Meanwhile, there was significant bifurcation of high-level fluconazole resistance into two distinct genomic pathways. Unlike Clade I and III, where resistance is predominantly driven by specific point mutations in the drug target *ERG11* (e.g., Y132F, K143R), the resistant isolates in this Clade II cohort lacked any coding mutations in the *ERG11* locus [9,18,22]. Fluconazole resistance was driven by either the seven-enzyme “FRM” network or a cryptic non-coding mechanism. This departure from the classic *ERG11*-centric model suggests that Clade II isolates have accessed alternative compensatory pathways to adapt to survival under azole stress, which potentially involves the modulation of membrane dynamics or efflux pump regulation. In the FRM subclade, fluconazole resistance was strongly correlated with the stepwise accumulation of mutations in a seven-gene FRM network, which supports the “dosage-dependent” evolutionary model (R = 0.69). The identification of a specific accumulation trajectory (0 → 5 → 6 → 7) suggests that the core five-gene complex (*TAC1B, PMA1, NIC96, EXO70, PCK1*) serves as a prerequisite “evolutionary ratchet,” stabilizing the cell against basal azole stress and creating a permissive environment for the subsequent acquisition of *CAN2* and *VPS53* mutations.

Structural validation was critical for the differentiation of functional drivers within this FRM network from neutral hitchhikers. The identification of *TAC1B* (F214S) as a primary driver aligns with previous reports linking *TAC1B* mutations to the constitutive overexpression of CDR1 efflux pumps [23,39]. However, the structural implication of the F214S substitution—creating a hydrophobic void at the dimerization interface—provides a refined mechanistic explanation for this loss-of-repressor function. Beyond transcriptional regulation, the validation of *PMA1* (E632D) and *NIC96* (L653F) suggests that Clade II resistance also relies on the alteration of membrane proton gradients and nuclear transport efficiency. The “shortening defect” in *PMA1* likely disrupts pH homeostasis, a known factor in fungal stress adaptation, while the steric hindrance in *NIC96* could selectively filter the nuclear import of stress-response transcription factors [41,42,43,54,55]. Furthermore, the *CAN2* (S198F) mutation introduces a bulky phenylalanine residue into the transporter pore, which generates severe steric clashes that likely occlude the channel and impair nutrient uptake, thereby triggering stress-response-mediated resistance [57,58]. Conversely, the classification of variants in *EXO70*, *PCK1*, and *VPS53* as “hitchhikers” highlights the importance of multi-tier structural filtering to avoid false-positive associations in genomic association studies [46,50]. Interestingly, while the clinical isolates exhibiting high-level fluconazole resistance remained clinically ‘susceptible’ to other azoles (itraconazole MICs ≤ 1 µg/mL and voriconazole/posaconazole ≤ 4 µg/mL), a detailed analysis of MIC values revealed a distinct cross-resistance trend (Table 1). The average MICs for voriconazole, itraconazole, and posaconazole increased in high-level fluconazole-resistant strains compared to the susceptible baseline, showing approximately a 12-fold increase for voriconazole (1.07 vs. 0.09 µg/mL), a 3-fold increase for itraconazole (0.29 vs. 0.11 µg/mL), and a 4-fold increase for posaconazole (0.20 vs. 0.05 µg/mL). This suggests that the identified resistance mechanisms for fluconazole also affected susceptibility to other members of the azole class. However, fluconazole lacks the extended hydrophobic side chains found in later-generation azoles (e.g., voriconazole, posaconazole), which stabilize drug–target interactions [60,61]. Therefore, genomic mechanisms often impact fluconazole resistance first because of its smaller structure. Larger azoles bind more tightly to a fungal target, which allows them to remain effective at standard doses even when fluconazole resistance is observed [16,62].

On the other hand, three mystery strains reportedly exhibited high-level resistance (MIC ≥ 128 μg/mL) despite a wild-type coding genotype in the validated FRM network. The complete lack of unique coding mutations in these strains strongly implies that non-coding regulatory mechanisms could be involved as an alternative mechanism of fluconazole resistance in Clade II isolates. Potential drivers could include promoter mutations or epigenetic modifications that upregulate efflux pumps (e.g., MDR1, CDR1) or alter sterol metabolism without changing the protein sequence [21,59]. This “stealth” resistance mechanism poses a significant diagnostic challenge, as it would be undetectable by standard targeted sequencing panels focused on *ERG11* or *TAC1B* coding regions. Future transcriptomic or methylome analyses can be used to unravel the precise regulatory nodes driving this phenotype. It is acknowledged that the functional impact of the identified mutations in this study was validated via high-confidence in silico modeling rather than in-vitro mutagenesis. However, the strict convergence of these mutations in the resistant phenotype, combined with the rigorous biophysical criteria applied (stability, conservation, proximity), provides compelling evidence for their causality. Additionally, while the sample size was limited to South Korean isolates, the inclusion of global reference strains ensures that these findings are phylogenetically relevant to the broader Clade II population.

## 5. Conclusions

In this study, 46% of Clade II strains demonstrated fluconazole resistance, with nine strains displaying a high level of resistance. The comprehensive three-level comparative genomic strategy revealed that this cohort does not rely on the classical *ERG11* mutations that are commonly observed in Clade I strains. Instead, two divergent trajectories were observed to be the genomic mechanism behind fluconazole resistance in high-level fluconazole-resistant mutants in this cohort: (1) a canonical, dosage-dependent accumulation of a seven-gene network of mutated genes driven by four key functional drivers: *TAC1B, PMA1, NIC96*, and *CAN2*, or (2) cryptic, non-coding mechanism that confers resistance without alteration of the coding genome. The delineation of the stepwise accumulation of (0 → 5 → 6 → 7) mutations in the FRM clade provides a potential biomarker panel for surveillance or novel antifungal drug targets, which could allow for the early detection and elimination of evolving antifungal resistance, before it reaches the maximal levels. However, the discovery of the “mystery” lineage highlights a critical blind spot in current genomic surveillance. As *C. auris* continues to adapt to clinical antifungal pressure, diagnostic strategies must expand beyond standard target gene sequencing to encompass regulatory elements and non-canonical resistance networks. These findings underscore the plasticity of the *C. auris* genome and the urgent need for constant vigilance in monitoring the molecular evolution of the East Asian Clade II lineage.

## Figures and Tables

**Table 1:** Antifungal susceptibility testing of *C. auris* according to CLSI guidelines.

Antifungal Agent	No. of Isolates with MIC (μg/mL) (Percentage of Isolates)														Resistance (%)
	≤0.015	0.03	0.06	0.125	0.25	0.5	1	2	4	8	16	32	64	≥128	
Fluconazole	-	-	-	-	-	-	-	2	9	6	4	7	3	9	46
Itraconazole	1	7	7	8	13	3									
Posaconazole	7	12	4	11	4	1									
Voriconazole	4	9	7	3	4	7	3	2							
Amphotericin B					7	23	8	1							
Flucytosine			15	15	8	1									
Caspofungin		3	13	22	1										
Anidulafungin	1	3	2	25	6	2									
Micafungin		9	14	15		1									

**Table 2:** Functional annotation of the 7-gene Clade II resistance network.

Gene	Mutation	Reference Strain	Protein Description		Reference
		*C. auris* B8441	Category	General Function	
*TAC1B*	F214S	B9J08_004820	Transcriptional factor	Regulates the activity of drug-responsive efflux proteins, CDR1 and CDR2	[23,39,40]
*PMA1*	E632D	B9J08_002855	Ion pump (H^+^-ATPase) in plasma membrane	Involved in pH regulation and hyphal formation in the cytoplasm	[41,42,43]
*VPS53*	P725A	B9J08_001273	Component of the GARP complex	Involved in mediation of endosome-to-vacuole trafficking and vacuolar protein sorting; maintains sphingolipid homeostasis, which is required for membrane structure and maintenance	[44,45]
*EXO70*	S109F	B9J08_004869	Component of the Exocyst complex	involved in exocytosis; contributes to the fusion and anchoring of secretory organs and transport vesicles to the plasma membrane through the interaction between GTPase Rho	[46,47,48,49]
*PCK1*	E487D	B9J08_002669	Phosphoenolpyruvate carboxykinase	Key enzyme in gluconeogenesis, where it converts oxaloacetate to phosphoenolpyruvate	[50,51,52,53]
*NIC96*	L653F	B9J08_003048	Nuclear pore complex (NPC) component	Regulates stress response gene expression by controlling nuclear import/export of transcription factors	[54,55]
*CAN2*	S198F	B9J08_005360	Neutral amino acid permease in plasma membrane	Modulates nutrient uptake and pH homeostasis, influencing stress responses; contributes to biofilm formation	[56,57,58,59]

**Table 3:** Multi-tier in silico structural validation of candidate mutations for classification of the seven network mutations as “functional drivers” or “hitchhikers”. The symbols (✅) and (❌) indicate whether the mutation satisfied (“Pass”) or failed to satisfy (“Fail”) the specified biophysical or evolutionary criterion.

Gene (Mutation)	Stability (ΔΔG)	Conservation	Proximity (<10 Å)	Surface Exposure	Interface Impact	Total Score	Final Classification
*TAC1B* (F214S)	−3.81 kcal/mol (Destabilizing) ✅ Pass	98.5% ✅ Pass	3.1 Å (Direct) ✅ Pass	Buried ❌ Fail	Dimer Interface ✅ Pass	4/5	Functional Driver
*NIC96* (L653F)	−1.69 kcal/mol (Destabilizing) ✅ Pass	95.0% ✅ Pass	12.0 Å ❌ Fail	Exposed ✅ Pass	Nup Complex ✅ Pass	3/5	Functional Driver
*PMA1* (E632D)	−0.43 kcal/mol Neutral ❌ Fail	97.2% ✅ Pass	8.5 Å Proximal ✅ Pass	Exposed ✅ Pass	❌ Fail	3/5	Functional Driver
*CAN2* (S198F)	−0.38 kcal/mol Neutral ❌ Fail	94.3% ✅ Pass	15.2 Å Pore Channel ✅ Pass	Exposed ✅ Pass	❌ Fail	3/5	Functional Driver
*EXO70* (S109F)	−0.32 kcal/mol Neutral ❌ Fail	96.1% ✅ Pass	10.7 Å ❌ Fail	Exposed ✅ Pass	❌ Fail	2/5	Hitchhiker
*PCK1* (E487D)	−0.17 kcal/mol Neutral ❌ Fail	93.8% ✅ Pass	18.4 Å ❌ Fail	Exposed ✅ Pass	❌ Fail	1/5	Hitchhiker
*VPS53* (P725A)	−0.05 kcal/mol Neutral ❌ Fail	92.5% ✅ Pass	16.8 Å ❌ Fail	Exposed ✅ Pass	❌ Fail	2/5	Hitchhiker

## Data Availability

The genomic sequencing data presented in this study are openly available in the NCBI BioProject database (https://www.ncbi.nlm.nih.gov/bioproject/PRJNA1343880) under accession number PRJNA1343880. However, detailed patient clinical metadata presented in this study are available upon request from the corresponding author, due to ethical restrictions.

## Supplementary Materials

The following supporting information can be downloaded at https://www.mdpi.com/article/10.3390/jof12010076/s1, Supplementary Table S1: Clinical and genomic metadata of fluconazole-resistant and -susceptible Clade II *Candidozyma auris* isolates.
